# Supplementary material for: Mechanistic insight into the role of AUXIN RESISTANCE4 in trafficking of AUXIN1 and LIKE AUX1-2
Source: Plant Physiol. 2023 Sep 30;194(1):422–33. doi: 10.1093/plphys/kiad506 (PMC10756756; doi:10.1093/plphys/kiad506)
Supplement: kiad506_Supplementary_Data [file kiad506_supplementary_data.zip › Suppemental Dataset S1/5445e4f6eaa2385e-AtAXR4/aligs/d2b61a1.179.alig.html]

Phyre 2 alignment of AtAXR4\_\_\_ with d2b61a1


|  |  |
| --- | --- |
| Return to main results | Retrieve Phyre Job Id |


|  |  |  |  |
| --- | --- | --- | --- |
| Job Description | AtAXR4\_\_\_ | | |
| Confidence | 100.00% | Date | Tue Jan 10 14:09:39 GMT 2023 |
| Rank | 179 | Aligned Residues | 283 |
| % Identity | 13% | Template | d2b61a1 |


| SCOP info | alpha/beta-Hydrolases | alpha/beta-Hydrolases | O-acetyltransferase |
| Resolution | 1.65 | | |

Show / Hide SS confidence

Show / Hide Conservation and Alignment quality

|  |  |  |  |  |  |  |  |  |  |
| --- | --- | --- | --- | --- | --- | --- | --- | --- | --- |
|  | Insertion relative to template ||  | Deletion relative to template |
|  | Catalytic residue from the CSA |
|  | |
| Detailed help on interpreting your alignment | |

  
  

|  |  |  |  |  |  |  |  |  |  |  |  |  |  |  |  |  |  |  |  |  |  |  |  |  |  |  |  |  |  |  |  |  |  |  |  |  |  |  |  |  |  |  |  |  |  |  |  |  |  |  |  |  |  |  |  |  |  |  |  |  |  |
| --- | --- | --- | --- | --- | --- | --- | --- | --- | --- | --- | --- | --- | --- | --- | --- | --- | --- | --- | --- | --- | --- | --- | --- | --- | --- | --- | --- | --- | --- | --- | --- | --- | --- | --- | --- | --- | --- | --- | --- | --- | --- | --- | --- | --- | --- | --- | --- | --- | --- | --- | --- | --- | --- | --- | --- | --- | --- | --- | --- | --- | --- |
|  |  | 92 | . | . | . | . | . | . | . | 100 | . | . | . | . | . | . | . | . | . | 110 | . | . | . | . | . | . |  |  |  | . | . | . | 120 | . | . | . | . | . | . | . | . | . | 130 | . | . | . | . | . | . | . | . | . |  |  |  |  |  |  |  |  |  |
| Predicted Secondary structure |  |  |  |  |  |  |  | --- | --- | --- | --- | --- | --- | --- |  |  |  |  |  |  |  | --- | --- | --- | --- | --- | . | . | . | --- | --- |  |  |  |  |  | --- | --- | --- | --- | --- | --- |  |  |  |  |  |  |  |  |  |  | . | . | . | . | . | . | . | . | . |
| Query SS confidence |  | --- | --- | --- | --- | --- | --- | --- | --- | --- | --- | --- | --- | --- | --- | --- | --- | --- | --- | --- | --- | --- | --- | --- | --- | --- | . | . | . | --- | --- | --- | --- | --- | --- | --- | --- | --- | --- | --- | --- | --- | --- | --- | --- | --- | --- | --- | --- | --- | --- | --- | . | . | . | . | . | . | . | . | . |
| Query Sequence |  | R | T | I | K | V | Q | V | N | S | N | E | S | P | I | E | V | F | V | A | E | S | G | S | I | H | . | . | . | T | E | T | V | V | I | V | H | G | L | G | L | S | S | F | A | F | K | E | M | I | Q | S | . | . | . | . | . | . | . | . | . |
| Query Conservation |  |  |  |  |  |  |  |  |  |  |  |  |  |  | --- |  |  |  |  |  | --- |  | --- |  |  |  | . | . | . |  |  | --- | --- | --- | --- | --- | --- | --- |  |  |  | --- | --- |  |  | --- | --- |  | --- |  |  |  | . | . | . | . | . | . | . | . | . |
| Template Conservation |  |  |  |  |  | --- |  |  | --- |  | --- |  |  |  |  |  | --- |  | --- |  |  |  | --- |  |  |  |  |  |  |  | --- |  | --- | --- |  | --- | --- | --- |  |  |  | --- |  |  |  |  |  |  |  |  |  |  |  |  |  |  |  |  |  |  |  |
| Template Sequence |  | Q | P | L | T | L | X | L | G | G | K | L | S | Y | I | N | V | A | Y | Q | T | Y | G | T | L | N | D | E | K | N | N | A | V | L | I | C | H | A | L | T | G | D | A | E | P | Y | F | D | D | G | R | D | G | W | W | Q | N | F | X | G | A |
| Template Known Secondary structure |  | S | --- |  |  | --- | T | T | S | --- |  |  | --- | S |  |  |  |  |  |  |  |  | S | --- | --- | --- | T | T | --- | --- | --- |  |  |  |  |  | --- | --- | T | T | --- | --- | S | --- | S | --- | --- | S | S | S | --- | --- | --- | T | T | G | G | G |  |  | T |
| Template Predicted Secondary structure |  | --- | --- |  |  |  | --- | --- | --- | --- |  |  | --- | --- |  |  |  |  |  |  |  | --- | --- | --- | --- | --- | --- | --- | --- | --- | --- |  |  |  |  |  | --- | --- | --- | --- | --- | --- |  |  |  |  |  |  |  | --- | --- | --- | --- | --- |  |  |  |  | --- | --- | --- |
| Template SS confidence |  | --- | --- | --- | --- | --- | --- | --- | --- | --- | --- | --- | --- | --- | --- | --- | --- | --- | --- | --- | --- | --- | --- | --- | --- | --- | --- | --- | --- | --- | --- | --- | --- | --- | --- | --- | --- | --- | --- | --- | --- | --- | --- | --- | --- | --- | --- | --- | --- | --- | --- | --- | --- | --- | --- | --- | --- | --- | --- | --- | --- |
|  |  | 12 | . | . | . | . | . | . | . | 20 | . | . | . | . | . | . | . | . | . | 30 | . | . | . | . | . | . | . | . | . | 40 | . | . | . | . | . | . | . | . | . | 50 | . | . | . | . | . | . | . | . | . | 60 | . | . | . | . | . | . | . | . | . | 70 | . |
|  |  |  |  |  |  |  |  |  |  |  |  |  |  |  |  |  |  |  |  |  |  |  |  |  |  |  |  |  |  |  |  |  |  |  |  |  |  |  |  |  |  |  |  |  |  |  |  |  |  |  |  |  |  |  |  |  |  |  |  |  |  |
|  |  |  |  |  | 140 | . | . | . | . | . | . | . | . | . | 150 | . | . | . | . | . | . | . | . | . | 160 | . | . | . | . | . | . | . | . | . | 170 | . | . | . | . | . | . | . | . | . | 180 | . | . | . | . | . | . | . | . | . | 190 | . | . | . | . | . | . |
| Predicted Secondary structure |  | . | . | . |  |  |  | --- | --- | --- |  |  |  |  |  | --- | --- | --- | --- | --- | --- | --- | --- | --- | --- | --- | --- | --- | --- | --- | --- | --- | --- | --- | --- | --- | --- | --- | --- | --- | --- | --- | --- |  |  |  | --- | --- | --- | --- | --- | --- | --- | --- | --- | --- | --- | --- | --- | --- | --- |
| Query SS confidence |  | . | . | . | --- | --- | --- | --- | --- | --- | --- | --- | --- | --- | --- | --- | --- | --- | --- | --- | --- | --- | --- | --- | --- | --- | --- | --- | --- | --- | --- | --- | --- | --- | --- | --- | --- | --- | --- | --- | --- | --- | --- | --- | --- | --- | --- | --- | --- | --- | --- | --- | --- | --- | --- | --- | --- | --- | --- | --- | --- |
| Query Sequence |  | . | . | . | L | G | S | K | G | I | H | S | V | A | I | D | L | P | G | N | G | F | S | D | K | S | M | V | V | I | G | G | D | R | E | I | G | F | V | A | R | V | K | E | V | Y | G | L | I | Q | E | K | G | V | F | W | A | F | D | Q | M |
| Query Conservation |  | . | . | . | --- | --- |  |  | --- |  | --- | --- | --- | --- |  | --- | --- | --- | --- |  | --- |  | --- |  |  | --- |  |  |  |  |  |  |  |  |  |  |  |  |  |  |  |  |  |  |  |  |  |  |  |  |  |  |  |  |  |  |  |  |  |  |  |
| Template Conservation |  |  |  |  | --- |  |  |  |  |  |  | --- | --- |  |  | --- |  |  | --- |  | --- |  | --- |  |  |  |  |  |  |  |  |  |  |  |  |  |  |  | . | . | . | . | . | . | . | . | . | . | . | . | . | . | . | . | . | . | . | . | . | . | . |
| Template Sequence |  | G | L | A | L | D | T | D | R | Y | F | F | I | S | S | N | V | L | G | G | C | K | G | T | T | G | P | S | S | I | N | P | Q | T | G | K | P | Y | . | . | . | . | . | . | . | . | . | . | . | . | . | . | . | . | . | . | . | . | . | . | . |
| Template Known Secondary structure |  | T | S | S |  |  | T | T | T | --- |  |  |  |  |  | --- | --- | T | T | --- | S | S | S | S | S | --- | T | T | S | B | --- | T | T | T | S | S | B | --- | . | . | . | . | . | . | . | . | . | . | . | . | . | . | . | . | . | . | . | . | . | . | . |
| Template Predicted Secondary structure |  | --- | --- | --- | --- | --- | --- | --- | --- | --- |  |  |  |  |  | --- | --- | --- | --- | --- | --- | --- | --- | --- | --- | --- | --- | --- | --- | --- | --- | --- | --- | --- | --- | --- | --- | --- | . | . | . | . | . | . | . | . | . | . | . | . | . | . | . | . | . | . | . | . | . | . | . |
| Template SS confidence |  | --- | --- | --- | --- | --- | --- | --- | --- | --- | --- | --- | --- | --- | --- | --- | --- | --- | --- | --- | --- | --- | --- | --- | --- | --- | --- | --- | --- | --- | --- | --- | --- | --- | --- | --- | --- | --- | --- | --- | --- | --- | --- | --- | --- | --- | --- | --- | --- | --- | --- | --- | --- | --- | --- | --- | --- | --- | --- | --- | --- |
|  |  | 72 | . | . | . | . | . | . | . | 80 | . | . | . | . | . | . | . | . | . | 90 | . | . | . | . | . | . | . | . | . | 100 | . | . | . | . | . | . | . | . |  |  |  |  |  |  |  |  |  |  |  |  |  |  |  |  |  |  |  |  |  |  |  |
|  |  |  |  |  |  |  |  |  |  |  |  |  |  |  |  |  |  |  |  |  |  |  |  |  |  |  |  |  |  |  |  |  |  |  |  |  |  |  |  |  |  |  |  |  |  |  |  |  |  |  |  |  |  |  |  |  |  |  |  |  |  |
|  |  | 197 | . | . | 200 | . | . | . | . | . | . | . | . | . | 210 | . | . | . | . | . | . | . | . | . | 220 | . | . | . | . | . | . | . | . | . | 230 | . | . | . | . | . | . | . | . | . | 240 | . | . | . |  | . | . | . | . | . | . | 250 | . | . | . | . | . |
| Predicted Secondary structure |  | --- | --- | --- | --- | --- | --- | --- | --- | --- | --- | --- | --- | --- | --- | --- | --- | --- | --- | --- | --- | --- | --- | --- | --- | --- | --- | --- | --- |  |  |  |  |  |  |  |  |  |  |  |  |  |  | --- | --- | --- | --- | --- | . |  |  |  |  |  |  | --- | --- |  |  |  |  |
| Query SS confidence |  | --- | --- | --- | --- | --- | --- | --- | --- | --- | --- | --- | --- | --- | --- | --- | --- | --- | --- | --- | --- | --- | --- | --- | --- | --- | --- | --- | --- | --- | --- | --- | --- | --- | --- | --- | --- | --- | --- | --- | --- | --- | --- | --- | --- | --- | --- | --- | . | --- | --- | --- | --- | --- | --- | --- | --- | --- | --- | --- | --- |
| Query Sequence |  | I | E | T | G | D | L | P | Y | E | E | I | I | K | L | Q | N | S | K | R | R | S | F | K | A | I | E | L | G | S | E | E | T | A | R | V | L | G | Q | V | I | D | T | L | G | L | A | P | . | V | H | L | V | L | H | D | S | A | L | G | L |
| Query Conservation |  |  |  |  |  |  |  |  |  |  |  |  |  |  |  |  |  |  |  |  |  |  |  |  |  |  |  |  |  |  |  |  |  | --- |  |  | --- |  |  |  | --- | --- |  | --- | --- | --- |  |  | . |  |  | --- | --- | --- | --- | --- |  | --- | --- |  | --- |
| Template Conservation |  | . | . | . | . | . | . | . | . | . | . | . | . | . | . | . | . | . | . | . | . |  |  |  |  |  |  | --- | --- |  |  | --- |  |  |  | --- | --- |  |  | --- | --- |  |  | --- | --- | --- |  |  |  |  |  | --- | --- | --- | --- | --- |  | --- | --- |  | --- |
| Template Sequence |  | . | . | . | . | . | . | . | . | . | . | . | . | . | . | . | . | . | . | . | . | G | S | Q | F | P | N | I | V | V | Q | D | I | V | K | V | Q | K | A | L | L | E | H | L | G | I | S | H | L | K | A | I | I | G | G | S | F | G | G | X | Q |
| Template Known Secondary structure |  | . | . | . | . | . | . | . | . | . | . | . | . | . | . | . | . | . | . | . | . | G | G | G | --- | --- | --- | --- | --- |  |  |  |  |  |  |  |  |  |  |  |  |  |  | T | T | --- | --- | --- |  |  |  |  |  |  |  | T |  |  |  |  |  |
| Template Predicted Secondary structure |  | . | . | . | . | . | . | . | . | . | . | . | . | . | . | . | . | . | . | . | . | --- | --- | --- | --- | --- | --- | --- | --- |  |  |  |  |  |  |  |  |  |  |  |  |  |  |  | --- | --- | --- | --- |  |  |  |  |  |  | --- | --- | --- |  |  |  |  |
| Template SS confidence |  | --- | --- | --- | --- | --- | --- | --- | --- | --- | --- | --- | --- | --- | --- | --- | --- | --- | --- | --- | --- | --- | --- | --- | --- | --- | --- | --- | --- | --- | --- | --- | --- | --- | --- | --- | --- | --- | --- | --- | --- | --- | --- | --- | --- | --- | --- | --- | --- | --- | --- | --- | --- | --- | --- | --- | --- | --- | --- | --- | --- |
|  |  |  |  |  |  |  |  |  |  |  |  |  |  |  |  |  |  |  |  |  |  | 109 | 110 | . | . | . | . | . | . | . | . | . | 120 | . | . | . | . | . | . | . | . | . | 130 | . | . | . | . | . | . | . | . | . | 140 | . | . | . | . | . | . | . | . |
|  |  |  |  |  |  |  |  |  |  |  |  |  |  |  |  |  |  |  |  |  |  |  |  |  |  |  |  |  |  |  |  |  |  |  |  |  |  |  |  |  |  |  |  |  |  |  |  |  |  |  |  |  |  |  |  |  |  |  |  |  |  |
|  |  | 256 | . | . | . | 260 | . | . | . | . | . | . | . | . | . | 270 | . | . | . | . | . | . | . | . | . | 280 | . | . | . | . | . | . | . |  |  |  |  |  |  |  |  |  |  |  |  |  |  |  |  |  |  |  |  |  |  |  |  |  |  |  |  |
| Predicted Secondary structure |  |  |  |  |  |  |  |  | --- |  |  |  |  | --- |  |  |  |  |  | --- | --- | --- | --- | --- | --- | --- | --- |  |  |  |  |  |  | . | . | . | . | . | . | . | . | . | . | . | . | . | . | . | . | . | . | . | . | . | . | . | . | . | . | . | . |
| Query SS confidence |  | --- | --- | --- | --- | --- | --- | --- | --- | --- | --- | --- | --- | --- | --- | --- | --- | --- | --- | --- | --- | --- | --- | --- | --- | --- | --- | --- | --- | --- | --- | --- | --- | . | . | . | . | . | . | . | . | . | . | . | . | . | . | . | . | . | . | . | . | . | . | . | . | . | . | . | . |
| Query Sequence |  | A | S | N | W | V | S | E | N | W | Q | S | V | R | S | V | T | L | I | D | S | S | I | S | P | A | L | P | L | W | V | L | N | . | . | . | . | . | . | . | . | . | . | . | . | . | . | . | . | . | . | . | . | . | . | . | . | . | . | . | . |
| Query Conservation |  | --- |  |  |  | --- |  |  |  | --- | --- | --- | --- |  |  | --- | --- | --- | --- | --- | --- |  |  |  |  |  |  | --- |  |  | --- |  |  | . | . | . | . | . | . | . | . | . | . | . | . | . | . | . | . | . | . | . | . | . | . | . | . | . | . | . | . |
| Template Conservation |  | --- |  |  |  | --- |  |  |  | --- |  |  | --- |  |  | --- | --- | --- |  |  |  |  |  |  |  |  |  |  |  |  |  |  |  |  |  |  |  |  |  |  |  |  |  |  |  |  |  |  |  |  |  |  |  |  |  |  |  |  |  |  |  |
| Template Sequence |  | A | N | Q | W | A | I | D | Y | P | D | F | X | D | N | I | V | N | L | C | S | S | I | Y | F | S | A | E | A | I | G | F | N | H | V | X | R | Q | A | V | I | N | D | P | N | F | N | G | G | D | Y | Y | E | G | T | P | P | D | Q | G | L |
| Template Known Secondary structure |  |  |  |  |  |  |  |  | S | T | T | S |  |  |  |  |  |  |  | S | --- | --- | S | S | --- | --- |  |  |  |  |  |  |  |  |  |  |  |  |  |  |  | T | S | T | T | --- | G | G | G | --- | --- | T | T | S | --- | --- | --- |  |  |  |  |
| Template Predicted Secondary structure |  |  |  |  |  |  |  |  |  |  |  |  | --- | --- |  |  |  |  |  | --- | --- | --- | --- | --- | --- | --- | --- | --- | --- |  |  |  |  |  |  |  |  |  |  |  |  | --- | --- |  |  |  |  |  |  |  |  | --- | --- | --- | --- | --- | --- |  |  |  |  |
| Template SS confidence |  | --- | --- | --- | --- | --- | --- | --- | --- | --- | --- | --- | --- | --- | --- | --- | --- | --- | --- | --- | --- | --- | --- | --- | --- | --- | --- | --- | --- | --- | --- | --- | --- | --- | --- | --- | --- | --- | --- | --- | --- | --- | --- | --- | --- | --- | --- | --- | --- | --- | --- | --- | --- | --- | --- | --- | --- | --- | --- | --- | --- |
|  |  | 149 | 150 | . | . | . | . | . | . | . | . | . | 160 | . | . | . | . | . | . | . | . | . | 170 | . | . | . | . | . | . | . | . | . | 180 | . | . | . | . | . | . | . | . | . | 190 | . | . | . | . | . | . | . | . | . | 200 | . | . | . | . | . | . | . | . |
|  |  |  |  |  |  |  |  |  |  |  |  |  |  |  |  |  |  |  |  |  |  |  |  |  |  |  |  |  |  |  |  |  |  |  |  |  |  |  |  |  |  |  |  |  |  |  |  |  |  |  |  |  |  |  |  |  |  |  |  |  |  |
|  |  |  |  |  |  |  |  |  |  |  |  | 288 | . | 290 | . | . | . | . | . | . | . | . | . | 300 | . | . | . | . | . | . | . | . | . | 310 | . | . | . | . | . | . | . | . | . | 320 | . | . | . | . | . | . | . | . | . | 330 | . | . | . | . | . | . | . |
| Predicted Secondary structure |  | . | . | . | . | . | . | . | . | . | . | --- |  |  |  |  |  |  |  |  |  | --- | --- |  |  |  |  |  |  |  |  |  |  | --- | --- | --- | --- | --- | --- |  |  |  |  |  |  |  |  |  |  |  |  | --- | --- | --- | --- |  |  |  |  |  |  |
| Query SS confidence |  | . | . | . | . | . | . | . | . | . | . | --- | --- | --- | --- | --- | --- | --- | --- | --- | --- | --- | --- | --- | --- | --- | --- | --- | --- | --- | --- | --- | --- | --- | --- | --- | --- | --- | --- | --- | --- | --- | --- | --- | --- | --- | --- | --- | --- | --- | --- | --- | --- | --- | --- | --- | --- | --- | --- | --- | --- |
| Query Sequence |  | . | . | . | . | . | . | . | . | . | . | V | P | G | I | R | E | I | L | L | A | F | S | F | G | F | E | K | L | V | S | F | R | C | S | K | E | M | T | L | S | D | I | D | A | H | R | I | L | L | K | G | R | N | G | R | E | A | V | V | A |
| Query Conservation |  | . | . | . | . | . | . | . | . | . | . |  |  |  |  |  |  |  |  |  |  |  |  |  |  |  |  |  |  | --- |  |  |  |  |  |  |  |  |  |  | --- |  | --- |  |  |  |  |  |  |  |  |  |  |  |  |  |  | --- |  |  |  |
| Template Conservation |  |  |  |  |  |  |  |  |  |  |  |  |  |  |  |  |  |  |  |  |  |  |  |  |  |  |  |  |  |  |  |  |  |  |  |  |  |  |  |  |  |  |  |  |  |  |  |  |  |  |  |  |  |  |  |  |  |  |  |  |  |
| Template Sequence |  | S | I | A | R | X | L | G | X | L | T | Y | R | T | D | L | Q | L | A | K | A | F | G | R | A | T | K | S | D | G | S | F | W | G | D | Y | F | Q | V | E | S | Y | L | S | Y | Q | G | K | K | F | L | E | R | F | D | A | N | S | Y | L | H |
| Template Known Secondary structure |  |  |  |  |  |  |  |  |  |  |  |  | S | --- |  |  |  |  |  |  |  | T | T | T | --- | B | --- | T | T | --- | --- | T | T | S | --- | --- | B |  |  |  |  |  |  |  |  |  |  |  |  |  |  | T | T | --- | --- |  |  |  |  |  |  |
| Template Predicted Secondary structure |  |  |  |  |  |  |  |  |  | --- | --- | --- | --- | --- |  |  |  |  |  |  |  |  |  |  |  |  | --- | --- | --- | --- | --- | --- | --- | --- | --- |  |  |  |  |  |  |  |  |  |  |  |  |  |  |  |  |  | --- | --- | --- |  |  |  |  |  |  |
| Template SS confidence |  | --- | --- | --- | --- | --- | --- | --- | --- | --- | --- | --- | --- | --- | --- | --- | --- | --- | --- | --- | --- | --- | --- | --- | --- | --- | --- | --- | --- | --- | --- | --- | --- | --- | --- | --- | --- | --- | --- | --- | --- | --- | --- | --- | --- | --- | --- | --- | --- | --- | --- | --- | --- | --- | --- | --- | --- | --- | --- | --- | --- |
|  |  | 209 | 210 | . | . | . | . | . | . | . | . | . | 220 | . | . | . | . | . | . | . | . | . | 230 | . | . | . | . | . | . | . | . | . | 240 | . | . | . | . | . | . | . | . | . | 250 | . | . | . | . | . | . | . | . | . | 260 | . | . | . | . | . | . | . | . |
|  |  |  |  |  |  |  |  |  |  |  |  |  |  |  |  |  |  |  |  |  |  |  |  |  |  |  |  |  |  |  |  |  |  |  |  |  |  |  |  |  |  |  |  |  |  |  |  |  |  |  |  |  |  |  |  |  |  |  |  |  |  |
|  |  | 338 | . | 340 | . | . | . |  |  |  | . | . | . | . | . | . | 350 | . | . | . | . | . | . | . | . | . | 360 | . | . | . | . | . | . | . | . | . | 370 | . | . | . | . | . | . | . | . | . | 380 | . | . | . | . | . | . |  |  |  |  | . | . | . | 390 |
| Predicted Secondary structure |  |  |  |  |  | --- | --- | . | . | . | --- | --- | --- | --- | --- |  |  |  |  |  |  |  | --- | --- | --- | --- | --- | --- |  |  |  |  |  | --- | --- | --- | --- | --- | --- | --- | --- |  |  |  |  |  |  |  |  |  |  | --- | --- | . | . | . | . | --- | --- |  |  |
| Query SS confidence |  | --- | --- | --- | --- | --- | --- | . | . | . | --- | --- | --- | --- | --- | --- | --- | --- | --- | --- | --- | --- | --- | --- | --- | --- | --- | --- | --- | --- | --- | --- | --- | --- | --- | --- | --- | --- | --- | --- | --- | --- | --- | --- | --- | --- | --- | --- | --- | --- | --- | --- | --- | . | . | . | . | --- | --- | --- | --- |
| Query Sequence |  | S | L | N | K | L | N | . | . | . | H | S | F | D | I | A | Q | W | G | N | S | D | G | I | N | G | I | P | M | Q | V | I | W | S | S | E | A | S | K | E | W | S | D | E | G | Q | R | V | A | K | A | L | P | . | . | . | . | K | A | K | F |
| Query Conservation |  |  |  |  |  |  |  | . | . | . |  |  |  |  |  |  |  |  |  |  |  | --- |  |  | --- |  | --- | --- | --- | --- | --- | --- | --- | --- |  |  | --- |  |  | --- |  |  |  |  | --- | --- |  | --- | --- |  |  | --- | --- | . | . | . | . |  | --- | --- |  |
| Template Conservation |  |  |  |  |  |  |  |  |  |  |  |  |  |  |  |  |  |  |  |  |  | --- |  |  | --- |  |  | --- | --- | --- | --- | --- |  | --- |  |  | --- |  |  |  | --- |  |  |  |  |  |  |  |  |  |  | --- |  |  |  |  |  |  |  |  |  |
| Template Sequence |  | L | L | R | A | L | D | X | Y | D | P | S | L | G | Y | E | N | V | K | E | A | L | S | R | I | K | A | R | Y | T | L | V | S | V | T | T | D | Q | L | F | K | P | I | D | L | Y | K | S | K | Q | L | L | E | Q | S | G | V | D | L | H | F |
| Template Known Secondary structure |  |  |  |  |  |  |  |  | --- | --- | T | T | T | T | S | S | --- |  |  |  |  |  | T | T | --- | --- | S |  |  |  |  |  |  |  | T | T | --- | S | S | S | --- |  |  |  |  |  |  |  |  |  |  |  |  |  | T | T | --- |  |  |  |  |
| Template Predicted Secondary structure |  |  |  |  |  |  |  |  |  | --- | --- | --- | --- | --- | --- | --- | --- |  |  |  |  |  |  | --- | --- | --- | --- | --- |  |  |  |  |  |  | --- | --- | --- | --- | --- | --- | --- |  |  |  |  |  |  |  |  |  |  |  |  | --- | --- | --- | --- | --- | --- |  |  |
| Template SS confidence |  | --- | --- | --- | --- | --- | --- | --- | --- | --- | --- | --- | --- | --- | --- | --- | --- | --- | --- | --- | --- | --- | --- | --- | --- | --- | --- | --- | --- | --- | --- | --- | --- | --- | --- | --- | --- | --- | --- | --- | --- | --- | --- | --- | --- | --- | --- | --- | --- | --- | --- | --- | --- | --- | --- | --- | --- | --- | --- | --- | --- |
|  |  | 269 | 270 | . | . | . | . | . | . | . | . | . | 280 | . | . | . | . | . | . | . | . | . | 290 | . | . | . | . | . | . | . | . | . | 300 | . | . | . | . | . | . | . | . | . | 310 | . | . | . | . | . | . | . | . | . | 320 | . | . | . | . | . | . | . | . |
|  |  |  |  |  |  |  |  |  |  |  |  |  |  |  |  |  |  |  |  |  |  |  |  |  |  |  |  |  |  |  |  |  |  |  |  |  |  |  |  |  |  |  |  |  |  |  |  |  |  |  |  |  |  |  |  |  |  |  |  |  |  |
|  |  | 391 | . | . | . | . |  |  | . | . | . | . | 400 | . | . | . | . | . | . | . | . | . | 410 | . | . | . | . | . | . | . |  |  |  |  |  |  |  |  |  |  |  |  |  |  |  |  |  |  |  |  |  |  |  |  |  |  |  |  |  |  |  |
| Predicted Secondary structure |  |  |  |  | --- | --- | . | . | --- | --- | --- | --- | --- | --- | --- | --- |  |  |  |  |  |  |  |  |  |  |  |  |  |  |  |  |  |  |  |  |  |  |  |  |  |  |  |  |  |  |  |  |  |  |  |  |  |  |  |  |  |  |  |  |  |
| Query SS confidence |  | --- | --- | --- | --- | --- | . | . | --- | --- | --- | --- | --- | --- | --- | --- | --- | --- | --- | --- | --- | --- | --- | --- | --- | --- | --- | --- | --- | --- |  |  |  |  |  |  |  |  |  |  |  |  |  |  |  |  |  |  |  |  |  |  |  |  |  |  |  |  |  |  |  |
| Query Sequence |  | V | T | H | S | G | . | . | S | R | W | P | Q | E | S | K | S | G | E | L | A | D | Y | I | S | E | F | V | S | L |  |  |  |  |  |  |  |  |  |  |  |  |  |  |  |  |  |  |  |  |  |  |  |  |  |  |  |  |  |  |  |
| Query Conservation |  | --- |  | --- |  | --- | . | . | --- | --- |  | --- | --- | --- | --- |  | --- |  | --- | --- | --- |  |  | --- |  | --- | --- | --- |  |  |  |  |  |  |  |  |  |  |  |  |  |  |  |  |  |  |  |  |  |  |  |  |  |  |  |  |  |  |  |  |  |
| Template Conservation |  |  |  | --- |  |  |  |  | --- | --- |  |  |  | --- | --- |  | --- | --- |  |  |  |  |  | --- |  |  | --- | --- | --- |  |  |  |  |  |  |  |  |  |  |  |  |  |  |  |  |  |  |  |  |  |  |  |  |  |  |  |  |  |  |  |  |
| Template Sequence |  | Y | E | F | P | S | D | Y | G | H | D | A | F | L | V | D | Y | D | Q | F | E | K | R | I | R | D | G | L | A | G |  |  |  |  |  |  |  |  |  |  |  |  |  |  |  |  |  |  |  |  |  |  |  |  |  |  |  |  |  |  |  |
| Template Known Secondary structure |  |  |  |  | --- | --- | T | T | G | G | G |  |  |  |  | --- |  |  |  |  |  |  |  |  |  |  |  |  |  | T |  |  |  |  |  |  |  |  |  |  |  |  |  |  |  |  |  |  |  |  |  |  |  |  |  |  |  |  |  |  |  |
| Template Predicted Secondary structure |  |  |  |  | --- | --- | --- | --- | --- | --- | --- |  |  |  |  |  |  |  |  |  |  |  |  |  |  |  |  |  | --- | --- |  |  |  |  |  |  |  |  |  |  |  |  |  |  |  |  |  |  |  |  |  |  |  |  |  |  |  |  |  |  |  |
| Template SS confidence |  | --- | --- | --- | --- | --- | --- | --- | --- | --- | --- | --- | --- | --- | --- | --- | --- | --- | --- | --- | --- | --- | --- | --- | --- | --- | --- | --- | --- | --- |  |  |  |  |  |  |  |  |  |  |  |  |  |  |  |  |  |  |  |  |  |  |  |  |  |  |  |  |  |  |  |
|  |  | 329 | 330 | . | . | . | . | . | . | . | . | . | 340 | . | . | . | . | . | . | . | . | . | 350 | . | . | . | . | . | . | . |  |  |  |  |  |  |  |  |  |  |  |  |  |  |  |  |  |  |  |  |  |  |  |  |  |  |  |  |  |  |  |
|  |  |  |  |  |  |  |  |  |  |  |  |  |  |  |  |  |  |  |  |  |  |  |  |  |  |  |  |  |  |  |  |  |  |  |  |  |  |  |  |  |  |  |  |  |  |  |  |  |  |  |  |  |  |  |  |  |  |  |  |  |  |

|  |  |  |
| --- | --- | --- |
| Download: | Text version | FASTA version |

No model constructed - rank, confidence too low

  

  

---

Phyre is now FREE for commercial users!

All images and data generated by Phyre2 are free to use in any
publication with acknowledgement

Accessibility Statement

**Please cite:** The Phyre2 web portal for protein modeling, prediction and analysis

Kelley LA *et al.* *Nature Protocols*
10, 845-858 (2015)
[paper]
[Citation
link]

|  |  |
| --- | --- |
| © Structural Bioinformatics Group, Imperial College, London |  |
| Lawrence Kelley, Michael Sternberg |  |
| Disclaimer |  |
| Terms and Conditions |  |

Phyre2 is part of **Genome3D**
